# Isolating phyllotactic patterns embedded in the secondary growth of sweet cherry (*Prunus avium* L.) using magnetic resonance imaging

**DOI:** 10.1186/s13007-019-0496-7

**Published:** 2019-10-04

**Authors:** Mitchell Eithun, James Larson, Gregory Lang, Daniel H. Chitwood, Elizabeth Munch

**Affiliations:** 1 Department of Computational Mathematics, Science and Engineering, Michigan State University, East Lansing, MI 48824 USA; 2 Department of Horticulture, Michigan State University, East Lansing, MI 48824 USA; 3 Department of Mathematics, Michigan State University, East Lansing, MI 48824 USA

**Keywords:** Magnetic resonance imaging, Sweet cherry, Phyllotaxy, Parastichy, Thresholding

## Abstract

**Background:**

Epicormic branches arise from dormant buds patterned during the growth of previous years. Dormant epicormic buds remain just below the surface of trees, pushed outward from the pith during secondary growth, but maintain vascular connections. Epicormic buds can be activated to elongate into a new shoot, either through natural processes or horticultural intervention, to potentially rejuvenate orchards and restructure tree architecture. Because epicormic structures are embedded within secondary growth, tomographic approaches are a useful method to study them and understand their development.

**Results:**

We apply techniques from image processing to determine the locations of epicormic vascular traces embedded within secondary growth of sweet cherry (*Prunus avium* L.), revealing the juvenile phyllotactic pattern in the trunk of an adult tree. Techniques include the flood fill algorithm to find the pith of the tree, edge detection to approximate the radius, and a conversion to polar coordinates to threshold and segment phyllotactic features. Intensity values from magnetic resonance imaging (MRI) of the trunk are projected onto the surface of a perfect cylinder to find the locations of traces in the “boundary image”. Mathematical phyllotaxy provides a means to capture the patterns in the boundary image by modeling phyllotactic parameters. Our cherry tree specimen has the conspicuous parastichy pair (2,3), phyllotactic fraction 2/5, and divergence angle of approximately 143°.

**Conclusions:**

The methods described provide a framework not only for studying phyllotaxy, but also for processing of volumetric image data in plants. Our results have practical implications for orchard rejuvenation and directed approaches to influence tree architecture. The study of epicormic structures, which are hidden within secondary growth, using tomographic methods also opens the possibility of studying genetic and environmental influences such structures.

## Background

Plants increase in length from apical meristems during primary growth. Located at the shoot tip, the shoot apical meristem is the site of cell division that produces leaf and bud primordia. Newly divided cells elongate, pushing the apical meristem upward. As the shoot continues to grow, leaf primordia cells divide, differentiate, and expand into leaves that subtend axillary buds. The point at which each leaf and axillary bud is attached to the shoot constitutes a node. In sweet cherry (*Prunus avium* L.), leaves and axillary buds form at each node in the year that a shoot develops. In non-juvenile plants, the axillary buds at nodes at the base of the shoot may differentiate into solitary flower buds. These bloom, develop fruit if pollinated and fertilized, and upon abscission of the fruit, the node becomes void of apparent vegetative or reproductive buds for subsequent growth. These are called “blind” nodes. The remaining non-basal, majority of nodes on the new shoot form a single vegetative bud at each node. In spring of the year after the shoot’s formation, each of these buds usually produces five to eight leaves that arise from very closely-packed nodes, producing a spur (a short, modified branch) along the rest of the length of the shoot, except for the terminal shoot apical meristem that again elongates to form a new section of shoot. Buds on some of the uppermost nodes of the original shoot also may elongate into new lateral shoots, rather than form spurs [1]. In the orchard, trees are considered mature once they have filled their allotted orchard space and have all of their reproductive components; in modern, high density plantings, this is 3 to 5 years.

The shoot apical meristem forms primordia in spiral patterns resembling cylindrical helices. *Phyllotaxy* is the study of the arrangement of plant organs during their development. For centuries, mathematicians have been interested in building models to describe the geometry of phyllotactic patterns [2, 3], using *phyllotactic parameters* to capture the layout and spacing of plant organs characteristic of different plant species and their stages of development [4]. These organs can be branches, leaves, vascular traces, or other features associated with primordia. Frequently, organ primordia form spiral patterns called *parastichies*, which can be characterized by Fibonacci-type sequences of numbers. In the *centric* representation of spiral phyllotaxy, parastichy spirals emanate from a central point, like the capitulum of a sunflower. In the *cylindrical* representation, parastichies are helices on the surface of a cylinder, like in pineapples or pinecones [2, 5]. It is convenient to unwrap the surface of the cylinder and view the primordia as a cylindrical lattice in the plane, called a *Bravais lattice*, in which intersections of parastichies are primordia. Previously, mathematical phyllotaxy has been used to predict the cylindrical arrangement of branches in Monterey pine (*Pinus radiata*) [6].

**Fig. 1:**
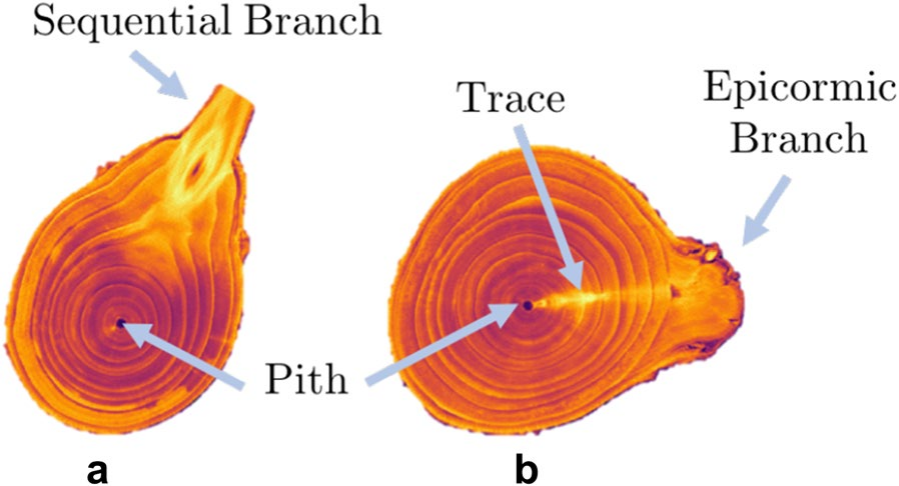
Anatomy of sweet cherry wood. Two labeled slices from the MRI scan of sweet cherry, colored by intensity value. The pith is the hollow center of the trunk. Slice (**a**) shows the annual growth rings growing towards the sequential branch. Slice (**b**) shows an epicormic branch that that had developed in the same year the trunk was collected following the bud trace outside of the annual growth rings. Note that branches and epicormic traces are high intensity regions

After a shoot has elongated and the phyllotaxy of the nodes patterned, woody plants undergo secondary growth to increase in girth. Axillary buds usually remain dormant in the year of initiation, but when the primary shoot is damaged or is growing extremely rapidly, the axillary bud may elongate into a new lateral shoot. Following the year of initiation, axillary buds that subsequently do not form lateral shoots, spurs, or flowers, thereby remaining dormant will eventually become engulfed by secondary growth of the stem and persist beneath the bark as an epicormic bud meristem [7, 8]. Epicormic bud meristem cells divide and elongate with radial growth, maintaining their presence just beneath the bark, and leaving a vascular trace back to the pith, shown in Fig. 1. Epicormic traces are 2–5 mm high [9] and occur perpendicular to the pith [7, 10]. Epicormic buds may sprout into epicormic branches following a stress such as fire [11], insect defoliation [12], wind damage [13], competition [14], or pruning [15]. If the primary epicormic meristem becomes damaged, it may split via the initiation of subtending secondary bud meristems. Epicormic buds can be activated to elongate into new shoots under the conditions noted above, and serve as a reserve of future potential shoot growth that can be used to rejuvenate trees by growers.

Image processing techniques have previously been used for dendrochronology, the study of tree rings and their features [16–18], and to manually locate rameal traces in oak trunks [10]. Semiautomatic feature identification on trees using image processing has been used for tree ring identification using the Sobel operator [19]. In this work, we isolate the patterning of epicormic traces embedded in secondary growth from a magnetic resonance imaging (MRI) scan of an 8 year old sweet cherry tree.

An image processing framework is implemented that finds the pith and radius of the trunk across slices. Using these dimensions, a polar coordinate conversion of each slice reveals x-ray dense regions corresponding to epicormic traces. A blob detection algorithm segments the epicormic features and the resulting phyllotactic parameters are estimated. Our work reveals the juvenile phyllotactic pattern of a sweet cherry tree embedded within 7 years of secondary growth. The study of epicormic features has implications for orchard rejuvenation, and the analysis methods presented provide an empirically based method to measure phyllotactic patterning and isolate anatomical features in plants.

## Materials and methods

### Plant material and magnetic resonance imaging (MRI)

**Fig. 2:**
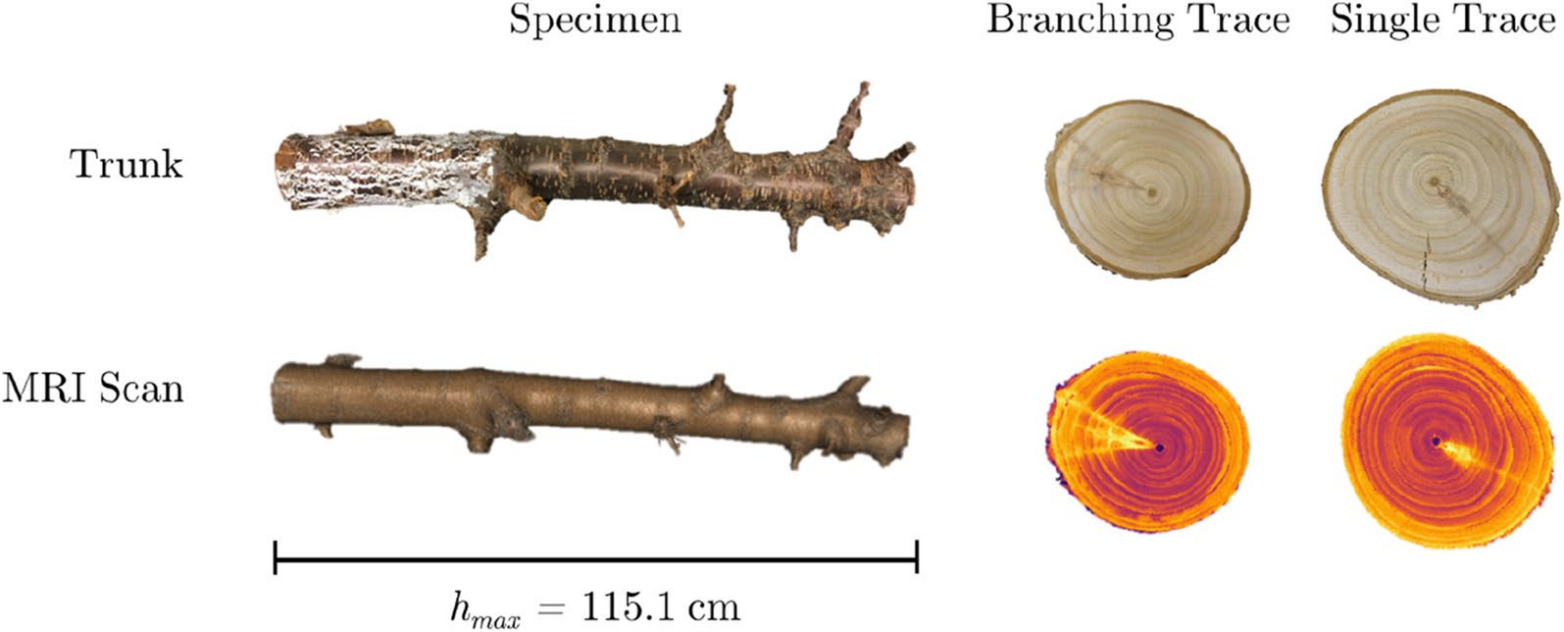
Cherry tree trunk and 3D reconstruction. MRI produces a voxel-based image of the specimen. Using 3DSlicer, an open-source software tool, we created a 3D reconstruction. Two types of traces (a branching trace and a single trace) are shown as physical cross-sections made by cutting the trunk and as slices from the scan

At bloom (late April) in 2016, the top of a 7-year-old sweet cherry tree, cultivar ‘NY 119’, planted at Michigan State University’s Clarksville Research Center and trained to a standard central leader canopy architecture, was removed with a chain saw 1.5 m from the ground. All lateral branches below that point were removed to promote the sprouting of epicormic buds along the remaining trunk length. Indeed, one of these epicormic buds was viable and sprouted during the 8th growing season, evidenced by a new branch that had emerged without disrupting the previous 7 years of secondary growth (see Fig. 1). Prior to and following this topping, the tree was managed with standard fertility, irrigation, and pest management procedures with the rest of the $\sim 0.25$ ha experimental orchard. In August, a 1.15 m-long section of the oldest portion of the tree’s trunk was fully removed just above the graft union and dried at room temperature in the laboratory. In December, this trunk section was scanned at slice thickness set of 0.625 mm, at Michigan State University’s Department of Radiology (East Lansing, MI) using a whole body magnetic resonance scanner (GE Signa HDX 3.0T, Chicago, IL). The scan took slightly over a minute and produced 1871 images, each of which is $250 \times 250$ pixels. Contrast in the scanned image is created by differences in moisture content; contrast is greater in air-dried than fresh wood [20]. The specimen and the scan are shown in Fig. 2. Table 1 gives estimates for the size and shape of the specimen.

**Table 1:** Sweet cherry specimen statistics

Measurement	Value
# MRI slices	1871
Tallest height	115.13 cm
Shortest height	110.31 cm
Median radius	4.72 cm ± 0.01 cm
Median circumference	29.64 cm ± 0.08 cm

Uncertainty is the standard error of the median

### Mathematical phyllotaxy

The goal of mathematical phyllotaxy is to describe emergent spiral patterns of lateral organs [21]. For the cylindrical representation, this includes viewing phyllotactic patterns as helices on a cylinder or sets of parallel lines, called parastichies.

**Fig. 3:**
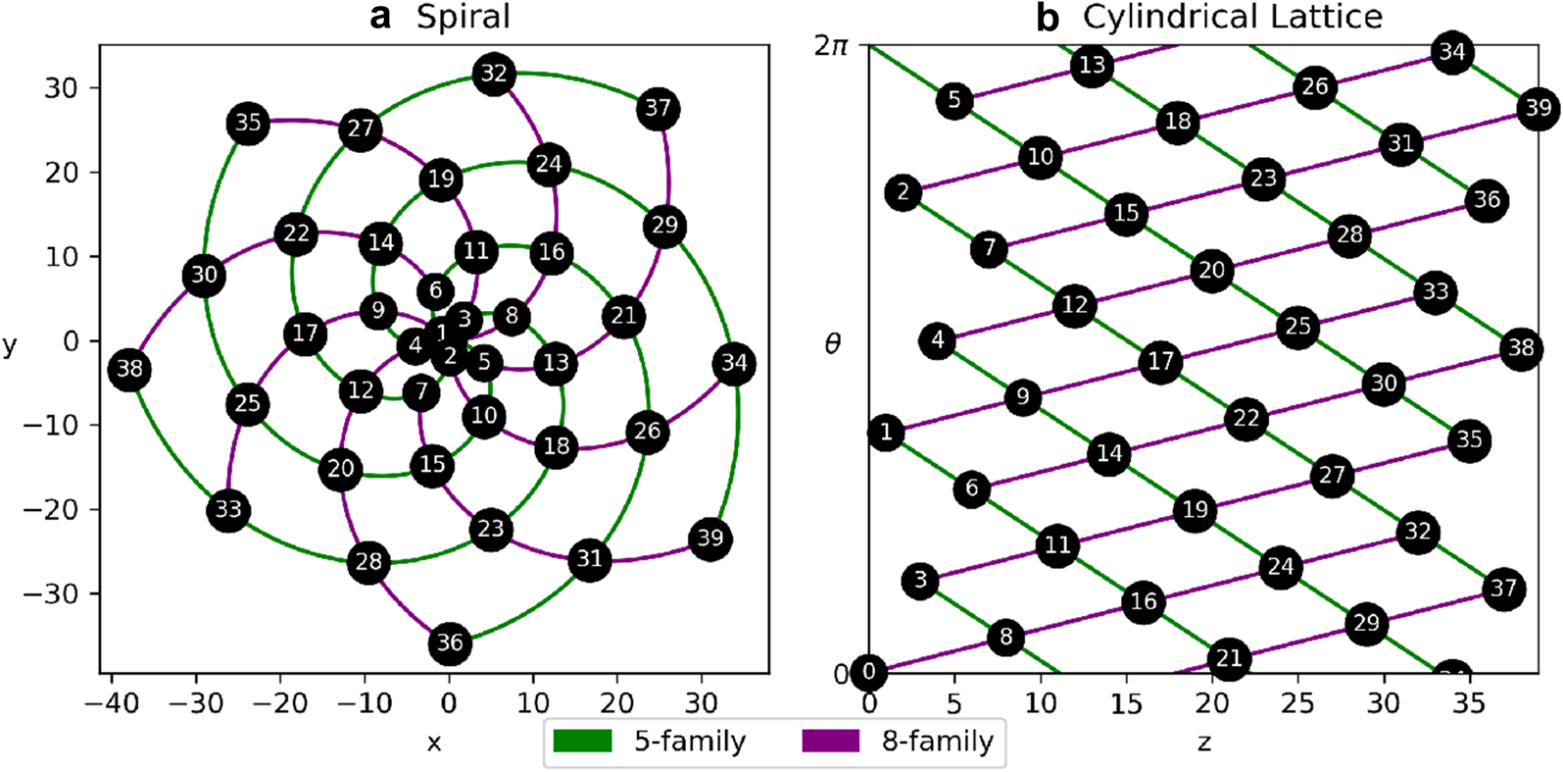
Two views of parastichy spirals from synthetic data. A set of 40 nodes, generated by the golden angle and constant rise ($r=1$). In the centric form, parastichies are spiral arms emanate from the origin and in the cylindrical form, parastichies are helices in a cylindrical lattice, called the Bravais lattice. In the Bravais lattice, the $\theta = 0$ and $\theta =2\pi$ rays are the same, allowing the parastichies to “wrap around” the circumference of the plant

Plant organs that comprise phyllotactic patterns are called *primordia*. In the case of our work, the primordia are epicormic buds near the epidermis connected by radial, vascular traces to the pith. A *parastichy* is a set of primordia that form a spiral arm. All parastichies that run in the same direction form a *family* and a parastichy in a family of *n* spirals is an *n-parastichy*. Figure 3 shows families of 5- and 8-parastichies in synthetic data. The 1-parastichy is called the *genetic spiral* and contains all primordia. A *contact parastichy* is one that is derived from the shape of the primordia. For example, each hexagonal primordia on the outside surface of a pineapple suggests three contact parastichies. Contact parastichies can be identified using Voronoi diagrams which tessellate the space around a set of points. Voronoi diagrams have been used in models of phyllotaxy using dynamical systems [3], mathematics to track the changing shape of primordia [22] and a geometric signature of a phyllotactic pattern [23].

A *parastichy pair* (*m*, *n*) is formed by *m* parastichies in one direction and *n* parastichies in the other. In *normal phyllotaxis*, parastichy pairs are often consecutive numbers in a Fibonacci-type sequence [2]. We restrict ourselves to the Fibonacci sequence $(F_n) = (1,2,3,5,8,\dots )$. A *visibly opposed* parastichy pair intersects only at primordia. There are many of these pairs in a phenomenon known as “rising phyllotaxy”. In order to choose a definite parastichy pair, we are interested in the *conspicuous parastichy pair*, which is a visibly opposed parastichy pair such that the angle of intersection between the two families is closest to 90° [2]. This pair is not necessarily unique, but it is the most “conscipuous” in the sense that the corresponding families of parastichies are the most noticeable.

#### Phyllotactic parameters

In cylindrical phyllotaxy, if there are *q* primordia, the coordinates of the primordia on the surface of the cylinder are given by

$$\begin{aligned} (z_0,\theta _0),(z_2,\theta _2),\dots ,(z_{q-1},\theta _{q-1}), \end{aligned}$$

where $\theta _i \in [0,2\pi )$ for $i=0,1,\dots ,m-1$ (this notation implies an arbitrary position for the ray $\theta =0$). The *divergence angle* *d* is defined as the average difference between successive angle measurements, i.e.

$$\begin{aligned} d :=\frac{1}{q-2} \sum _{i=1}^{q-2} \left[ (\theta _{i+1} - \theta _i) \text { mod } 2\pi \right] . \end{aligned}$$

For clarity we also define the *divergence fraction* as $d^* := d/2\pi$, which is the fraction of the angular breadth of the arc made by the divergence angle (in some literature this is the definition of the divergence angle [2]).

A useful parameter to complement the divergence angle is the vertical distance between successive primordia, or the *rise*, defined by the internode distance, $r_i := z_{i} - z_{i-1}$. The conspicuous parastichy pairs are a function of both the divergence angle and the rise. Hence, both parameters are necessary to fully to describe the phyllotaxy of the system.

Using the divergence angle *d*, idealized spiral nodes can be generated by $(x_n,y_n) = (n\cos {nd}, n\sin {nd})$ and cylindrical lattice points can be generated by $(z_n,\theta _n) = (n, (dn)/(2\pi ) \mod 2\pi )$. Examples of both centric and cylindrical phyllotaxy are shown in Fig. 3.

#### Phyllotactic fractions

In contrast to parastichy pairs, a more traditional way to describe a cylindrical phyllotactic system is to use a phyllotactic fraction to approximate the angular differences between nodes [24]. A *phyllotactic fraction* *A*/*B* (sometimes called “the phyllotaxis” of a species) is determined by the number of turns of a spiral, *A*, of successive leaves to reach the *B*th node directly above the starting node. In normal phyllotaxis *A* and *B* are numbers from a Fibonacci-type sequence such that there is exactly one term of the sequence in between *A* and *B*. For example, cherry’s phyllotactic fraction, derived from the Fibonacci sequence, is 2/5; this means that a node will have a node form above it after approximately two spirals of five successive nodes. A cherry stem will then have five vertical ranks of nodes called *orthostichies* [25]. The phyllotactic fraction is meant to approximate the divergence angle. For example, the phyllotactic fraction 2/5 implies a divergence angle of $d = (2/5){360}^{\circ } = {144}^{\circ }$.

#### Parastichy identification

Using the divergence fraction, we can identify parastichies in an idealized model describing the pattern of primordia. Theorem 1 helps identify which primordia belong to which parastichies. After sorting the primordia by their radial angle and then labelling the primordia by their position, we call two primordia *adjacent* if they are next to each other in this list.

##### Theorem 1

(The Bravais-Bravais Theorem, 1837 [2]) *On an* *n-parastichy of a phyllotactic spiral pattern, the numbers of two adjacent primordia differ by* *n*.

It follows from Theorem 1, for example, that the parastichies in the 2-parastichy family will have labels $\{0,2,4,\dots ,\}$ and $\{1,3,5,\dots ,\}$. In the case that of phyllotaxy based on the Fibonacci sequence, Theorem 2 characterizes parastichies that are visibly opposed. This Theorem is a specific case of the Fundamental Theorem of Phyllotaxy [2, 26]. For ease of notation let ${\text {nint}}(x)$ denote the nearest integer to the real number *x*.

##### Theorem 2

(Adler’s Theorem, 1974 [27]) *Let* $(F_k)_{k\in \mathbb {N}}$ *denote the Fibonacci sequence and define an interval*

$$\begin{aligned} I_k = {\left\{ \begin{array}{ll} {[}F_{k-2}/F_k, F_{k-1}/F_{k+1}], &{} k\,\, odd \\ {[}F_{k-1}/F_{k+1}, F_{k-2}/F_{k}], &{} k\,\, {even}. \end{array}\right. } \end{aligned}$$

*The parastichy pair* $(F_k,F_{k+1})$ *is visible and opposed if and only if* $d^* \in I_k$.

In other words, bounding the divergence angle in a particular way is necessary and sufficient to determine if a parastichy pair (*m*, *n*) is visible and opposed.

## Results

Using image processing techniques and mathematical phyllotaxy, we propose a method to algorithmically determine the conspicuous parastichy pair found in cylindrical phyllotaxy. This method could be the basis of an automatic method to retrieve the locations of primordia in 3D scans of plants with cylindrical phyllotaxy, given that the primordia are revealed by the distribution of intensity values. Again let ${\text {nint}}(x)$ denote the nearest integer to the real number *x*.

### Pre-processing

Mathematically, a $m \times n$ pixel slice (2D image) *Z* is a function

$$\begin{aligned} Z : \{0,1,\dots ,m-1\} \times \{0,1,\dots ,n-1\} \rightarrow [0,1]. \end{aligned}$$

The cherry tree scan is comprised of 1871 dicom files, each of which contains a $250 \times 250$ pixel slice. Each slice was taken parallel to the ground so that each is an image of rings of the tree at a fixed height. Since each slice represents a thickness of 0.625 mm and each pixel is 0.351562 mm $\times$ 0.351562 mm square each voxel cube in the image has a volume of about 0.077 mm. Using the pydicom Python package [28], we converted dicom files to numpy matrices [29].

### Pith finding algorithm

To identify the location of the pith in each slice, define a range of intensity values $C \subset [0,1]$, which represents the intensity values found in the pith in the whole scan. Suppose we know that pith is located at $(x_i,y_i)$ in slice $Z_i$. To find the pith in slice $Z_{i+1}$ we seed the breadth-first search (BFS) version of the flood fill algorithm with $(x_i,y_i)$ to find $(\hat{x},\hat{y})$, the nearest pixel with an intensity in *C* [30, p. 124]. Next we find the largest region containing of the image containing $(\hat{x},\hat{y})$ such that each pixel in the region has an intensity in *C*. The centroid of this region is the center of the pith in slice $Z_{i+1}$. We assume that the pith does not move significantly between slices so we mark the center of the pith in the first slice and use this algorithm to find the center of the pith in each slice.

### Radius estimation

**Fig. 4:**
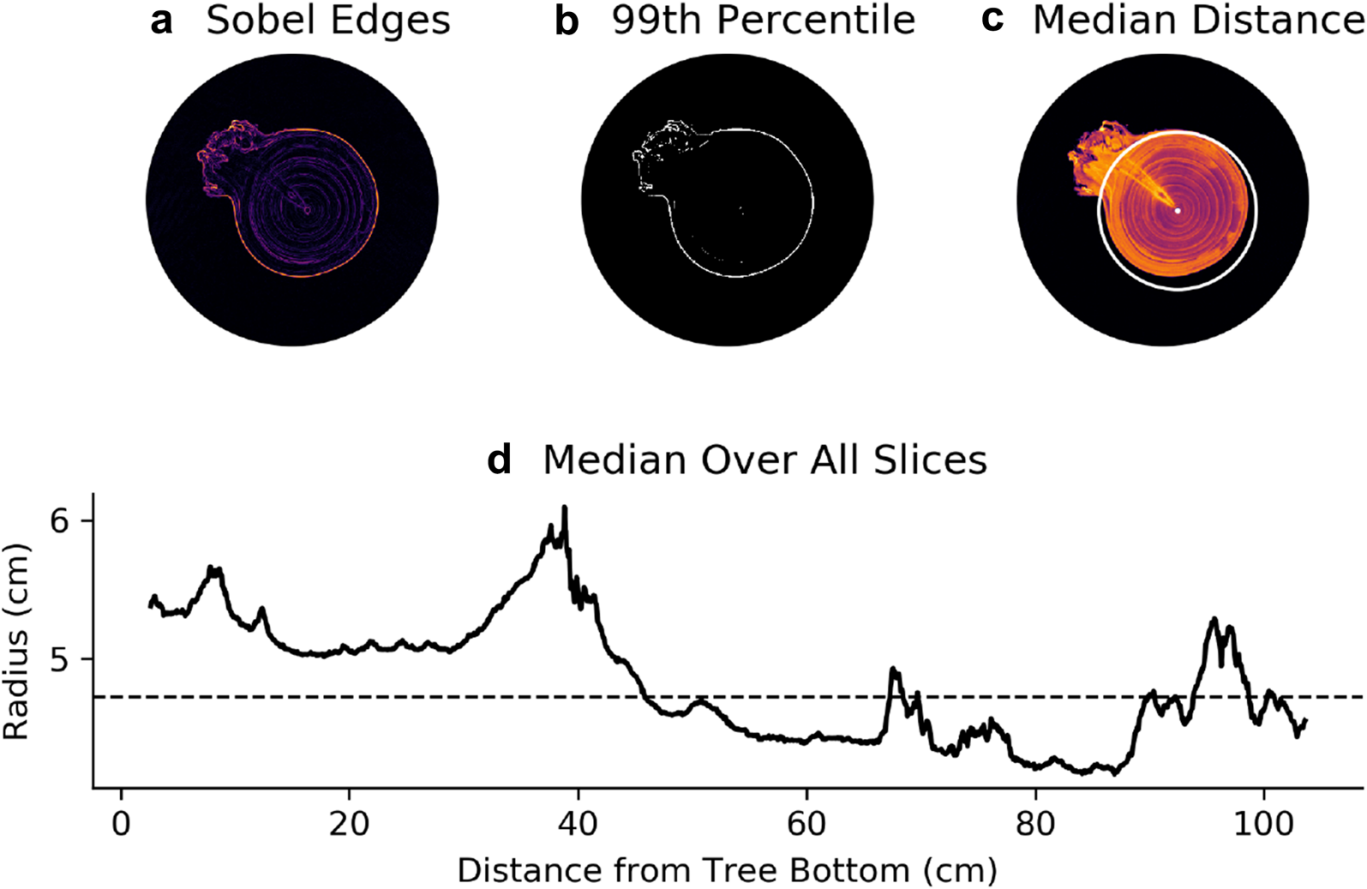
Algorithm to determine the approximate tree radius in each slice.** a** To estimate the radius of the trunk in each slice we first apply the Sobel Edge Detection algorithm to reveal areas of abrupt change in pixel intensity.** b** Then we threshold the image with $\alpha$ equal to the 99th percentile of the values given by edge detection.** c** Finally we compute the median distance from the pith location to all of the pixels in the binary image to estimate the radius. **d** To estimate the overall radius for the trunk, we compute the median of all radii for each slice. The dotted line represents the median

To estimate the average radius of the cherry tree we identify the edge of the tree in each slice and then compute the distance to the pith location. This process is outlined in Fig. 4. Specifically we apply the Sobel edge detection algorithm to identify probable edges [19, 31]. Since the edge detection algorithm also identifies rings and traces in the interior of the tree, we threshold the edge detection image and set all values below the 99th percentile to 0, which leaves values on the boundary of the tree. Then we compute the median distance from these points to the pith centroid to estimate the radius of a slice. Let $\rho _i$ denote the estimated radius of slice $Z_i$. We estimate the overall radius of the tree as the median of $\rho _i$ over all *i*.

### Conversion to polar coordinates

Using the coordinate pairs for pith locations and the estimated radii, we convert each slice to polar coordinates. Then the radial, wedged-shaped traces in the slices become vertical blocks and are easier to identify.

**Fig. 5:**
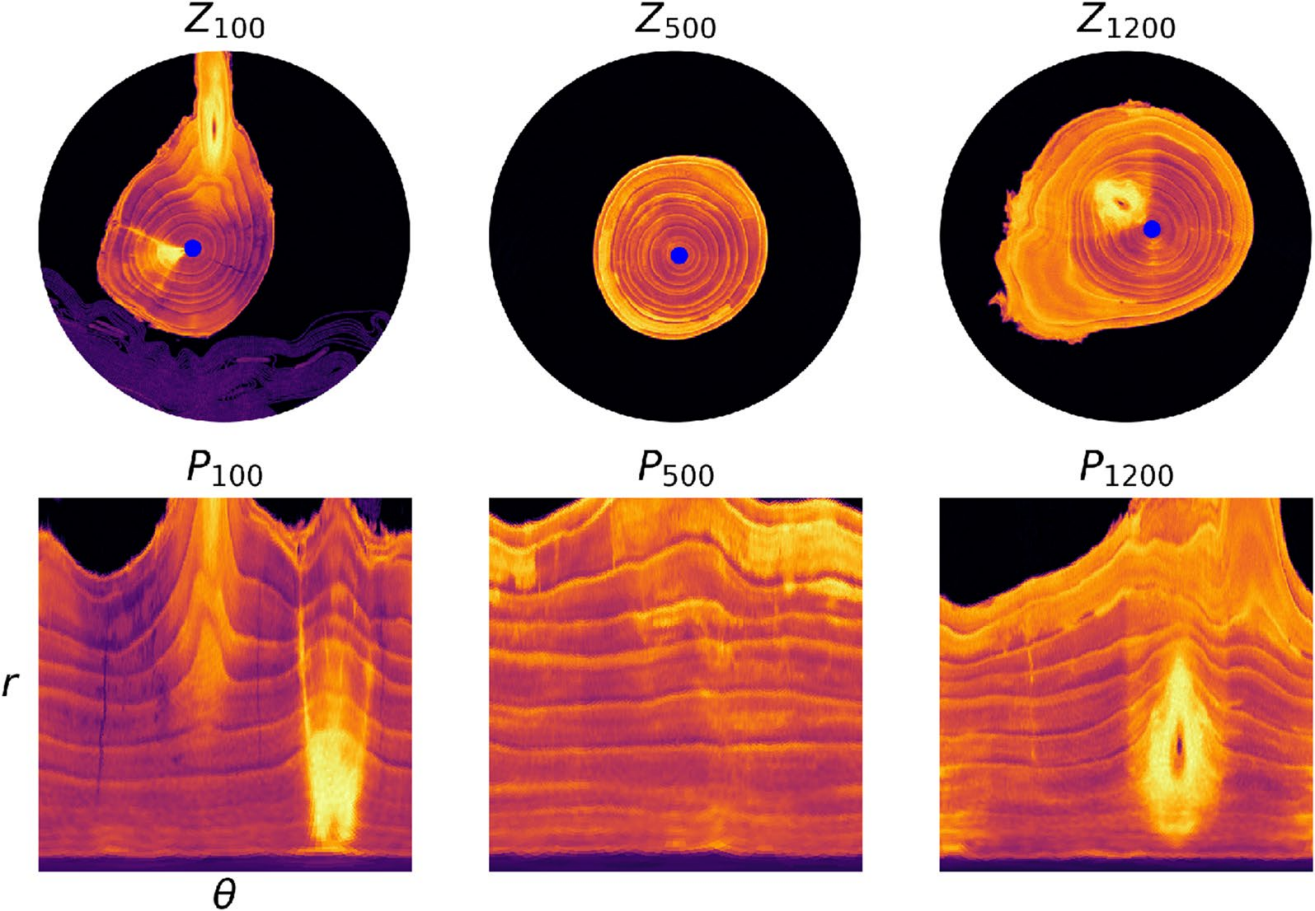
Polar coordinate conversion for selected slices. After finding the center and radius of each pixel slice $Z_i$ we convert to polar coordinates, producing polar slices $P_i$. The radius spans from 0 to the estimated radius $\rho _i$

Specifically, for each slice $Z_i$, define a *polar slice* $P_i$ by

$$\begin{aligned} P_i(r,\theta ) := Z_i \left({\text {nint}}( x_i + r\cos \theta ), \; {\text {nint}}(y_i + r\sin \theta) \right) \end{aligned}$$

for $\theta \in \{0,\alpha ,2\alpha ,\dots ,2\pi -\alpha \}, r \in \{0,s,2s,\dots ,\rho _i-s\}$, where $\alpha ,s$ are small positive numbers. Selected pixel slices and polar slices are shown in Fig. 5.

### Boundary image

Next we construct a radial summary of the polar slices that we call the *boundary image*. Notice that the traces and branches in Fig. 5 have higher intensity values than the other parts of the tree. This means the intensity values in columns of a polar image will where a radial trace is present. This is true for both branching traces and single traces (Fig. 2).

For each polar image $P_i$, define an intensity range $T_i \subset [0,1]$ that denotes the foreground of the image. Define the boundary image $\mathcal {B}:\{1,\dots ,N\} \times \{1,\dots ,\lfloor {\frac{2\pi }{\alpha }}\rfloor \} \rightarrow [0,1]$ by

$$\begin{aligned} \mathcal {B}(i, \theta ) := {\text {mean}}_r\{P_i(r,\theta ) \in T_i \}. \end{aligned}$$

Hence, the mean pixel intensity along columns in polar slices become rows in the boundary image (the intensity range $T_i$ restricts us to consider only pixels that are a part of the tree and not pixels that are in the background). Note that other summary statistics such as the median or the* p*th percentile may be used in place of the mean.

**Fig. 6:**
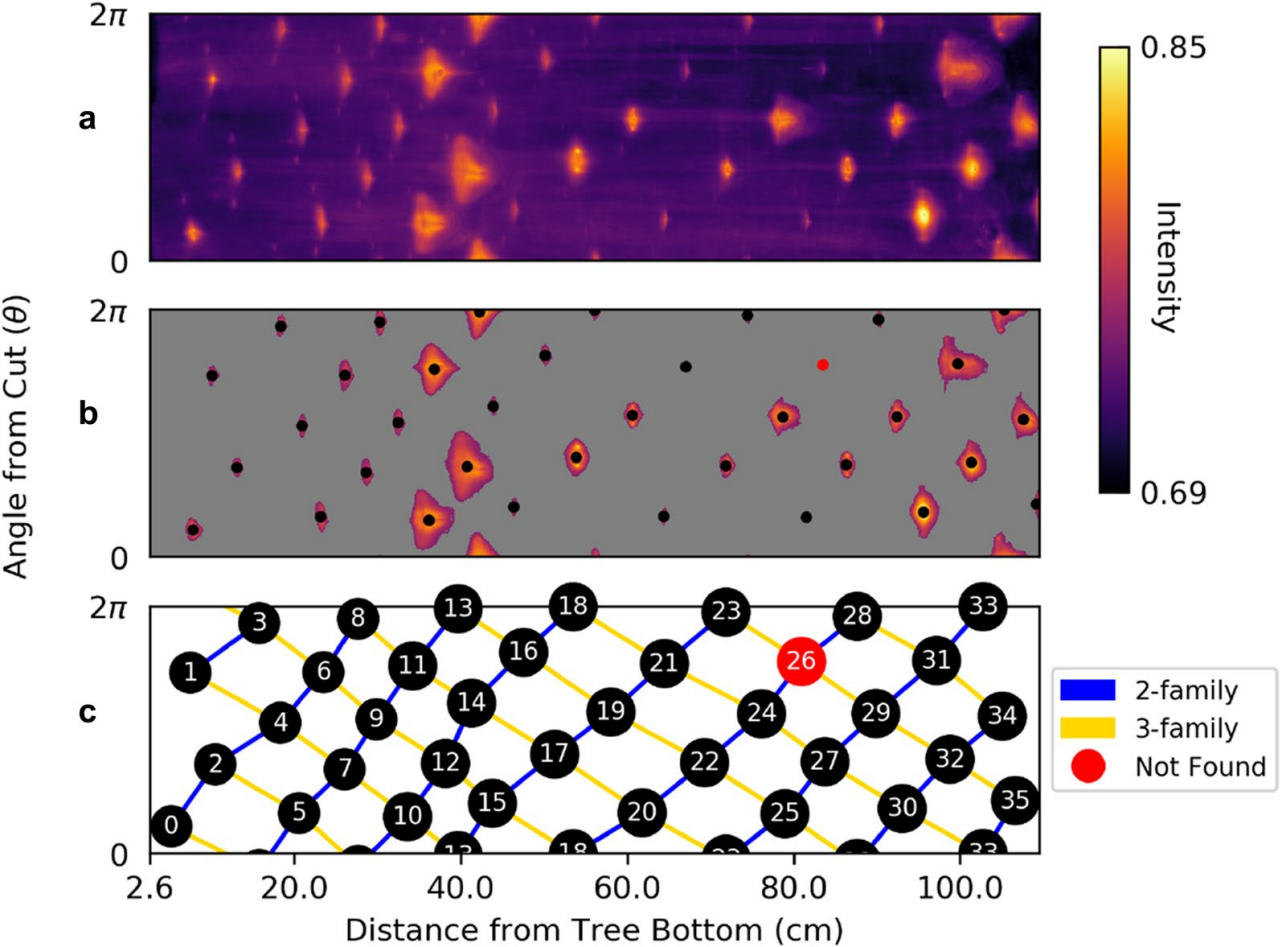
Isolating phyllotactic features in the boundary image.** a** The boundary image, created by assembling the means along each radial direction in the polar slices. Rows correspond to polar coordinates and columns slices.** b** The thresholded boundary image, created using Otsu’s method.** c** Cylindrical Bravais lattice of nodes, with 2- and 3-parastichies shown. The blob corresponding to node 26 was added by choosing a less restrictive threshold for that region

The boundary image is shown in Fig. 6a. The boundary rays $\theta = 0$ and $\theta = 2\pi$ glue together so that the boundary image is the boundary of a cylinder representing the cherry tree. Notice the phyllotactic patterns that emerge in the lattice structure of the image.

### Blob detection

Each blob in the boundary image corresponds to an epicormic trace in the cherry tree. To identify the centroid of each blob, we threshold the image using Otsu’s method [32]. Then we compute the centroid of each region to find the trace locations, shown in Fig. 6b. Notice that one blob (colored in red) was too small to be recognized. The centroid of this blob was found by using a less restrictive threshold and was added to the final dataset.

### Phyllotaxy parameter determination

Using the centroids of the blobs in Fig. 6b, we estimate phyllotactic parameters in the context of both the Adler’s Theorem (Theorem 2), phyllotactic fractions and Voronoi diagrams. A summary of phyllotactic parameters found for this sample is found in Table 2.

**Table 2:** Phyllotactic parameters with standard error

Parameter	Value
Number of primordia	36
Handedness	Counterclockwise
Divergence angle (*d*)	${142.928}^{\circ } \pm {1.181}^{\circ }$ (2.495 rad ± 0.021 rad)
Divergence fraction ($d^*$)	0.397 ± 0.003
Average rise (*r*)	2.896 cm ± 0.182 cm
Visible and opposed parastichy pairs	(1,2), (2,3), (3,5), (5,8)
Parastichy numbers	1, 2, 3, 5, 8
Conspicuous parastichy pair	(2,3)
Conspicuous phyllotactic fraction	$F_3/F_5 = 2/5$
Number of orthostichies	5

The “handedness” of the system is the direction of the genetic spiral starting from the bottom of the sample. The families of parastichies alternate between clockwise and counterclockwise direction

**Fig. 7:**
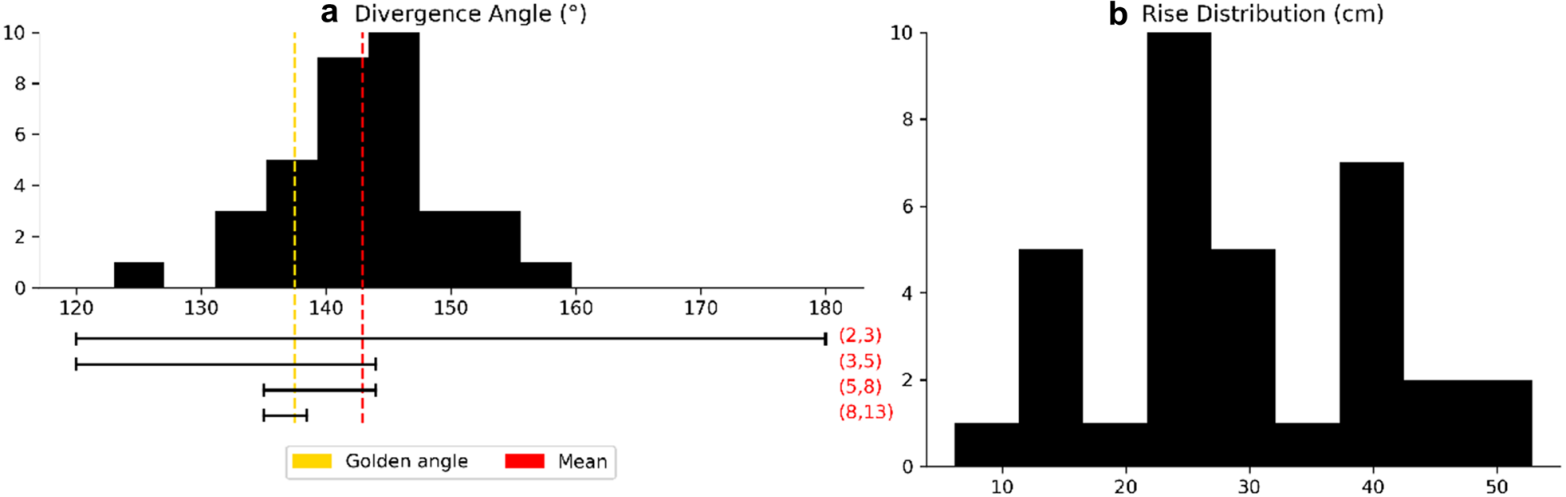
Distributions of phyllotactic parameters.** a** The distribution of local divergence angles compared to the golden angle ($\approx {137.5}^{\circ }$) and the mean ($\approx {139}^{\circ }$). The intervals given by Adler’s Theorem (Theorem 2) for visible and opposed parastichy pairs are shown below the distribution. Assuming the mean of the distribution is the true divergence angle, (1,2), (2,3), (3,5) and (5,8), are visible and opposed. The interval [60°, 180°], corresponding to the pair (1,2), is not shown.** b** The distribution of rise values, showing the range of internode lengths

**Fig. 8:**
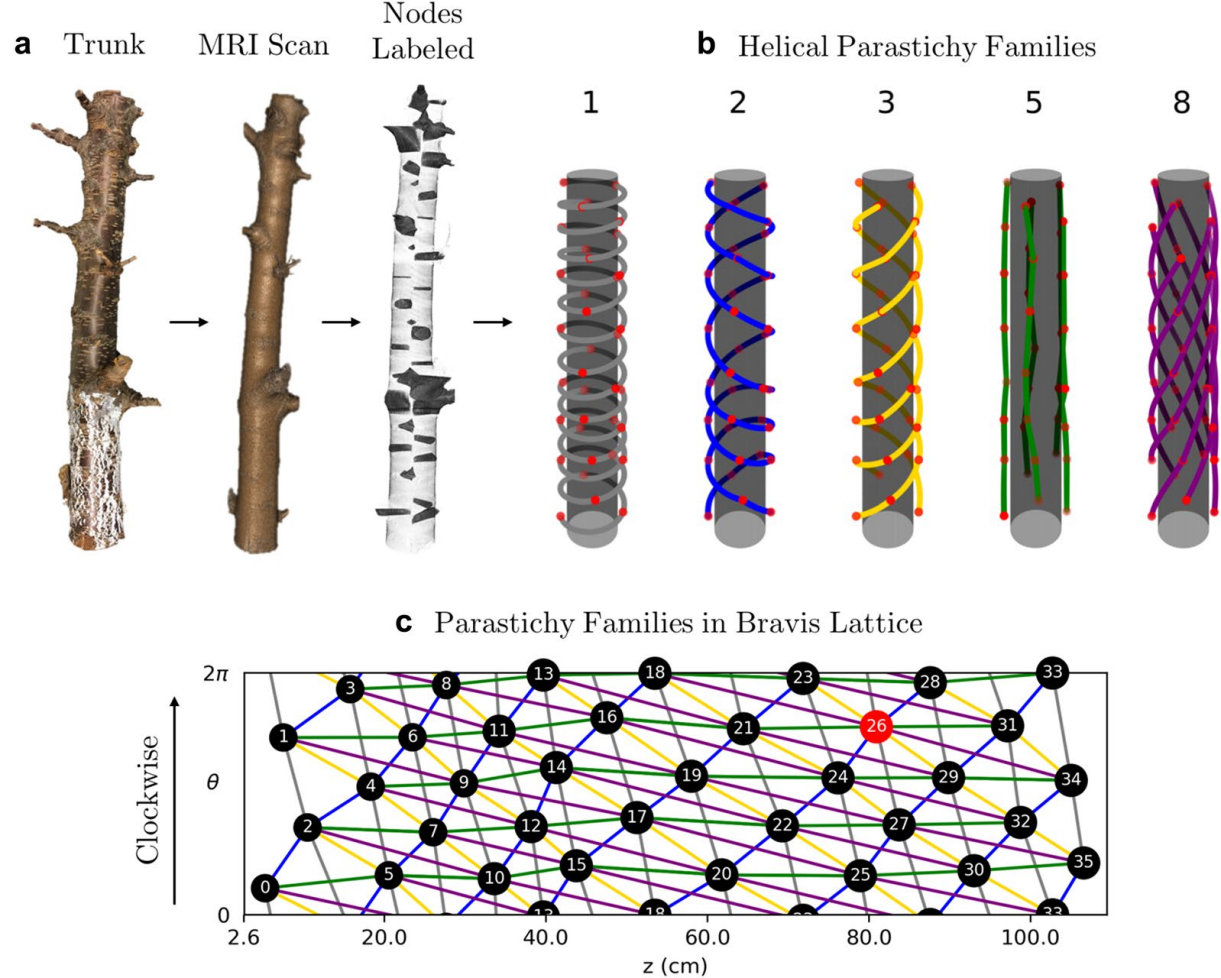
Visualizing parastichies in 3D. Families of parastichies using helical interpolation on a cylinder.** a** The blobs found in Fig. 6b map to wedge shaped regions in the MRI scan.** b** A 3D visualization of parastichy families that are a part of visible and opposed parastichy pairs.** c** Parastichy families shown in the Bravais lattice, with the same color scheme as part (**b**)

Start by sorting and labeling the 36 nodes by their *z* location so that node 0 is near the bottom of the trunk and node 35 is near the top. The divergence angle, estimated by the mean change in angle between successive primordia is $d = {142.928}^{\circ } \pm {1.181}^{\circ }$, which gives a divergence fraction of $d^* =$ 0.397 ± 0.003. To see the distribution of local divergence angles and rise, see Fig. 7. Assume that $d^*$ is the actual divergence fraction of the system. Since $d^* \in [3/8, 2/5]$, it follows from Adler’s Theorem that (1, 2), (2, 3), (3, 5), (5, 8) are all visible and opposed parastichy pairs. Also, 1, 2, 3, 5 and 8 are parastichy numbers in the sense that are part of visibly opposed parastichy pairs for the specimen. However, the standard error on $d^*$ suggests that the true mean could be greater than 2/5, which would exclude the 8-parastichies from consideration. Figure 8 shows all parastichies in 3D and on the Bravais lattice.

Since the second number in a phyllotactic fractions corresponds to a parastichy number, 1/2, 1/3, 2/5 and 3/8 are all phyllotactic fractions of the system (see [2, §2.2.2]). Traditionally, the phyllotactic fraction *A*/*B*, chosen to represent the phyllotaxy of a cylindrical system, is derived from the following observation: take two nodes whose labels differ by *A* and note that they are approximately above each other (having the same $\theta$ values) by going exactly *B* times around the genetic spiral. In this sense, the “phyllotaxy of the system” for the cherry specimen can be described as 2/5. We propose a more precise way of determining a single phyllotactic fraction: take the Fibonacci fraction closest to the divergence fraction $d^*$. In other words, for phyllotaxy based on the Fibonacci sequence, let $F_{I}/F_{I+2}$ be the *conspicuous phyllotactic fraction*, where $I = {\text {arginf}}_i\,|(F_i/F_{i+2}) - d^*|$. In our case, $I = 3$, $F_3 = 2$ and $F_5=5$ since 2/5 is closest to $d^* = 0.397$.

To determine the conspicuous parastichy pair, we calculate angles between parastichies. Using Theorem. 1, draw $F_{i}$-parastichies and $F_{i+1}$-parastichies using linear interpolation and measure the angles at each intersection. The pair of $F_i$ and $F_{i+1}$ for which this angle is closest to 90° is the conspicuous parastichy pair (all parastichy families are shown in Fig. 8). Using the law of cosines, we compute the average intersection angles between families of parastichy pairs and convert each to the “small angle”: 77.91° for (2,3); 35.21° for (3,5); and 14.75° for (5,8). Thus, by definition, the parastichy conspicuous parastichy pair is $(F_2,F_3) = (2,3)$. The 2-family and 3-family of parastichies are shown in the Bravais lattice in Fig. 6c.

**Fig. 9:**
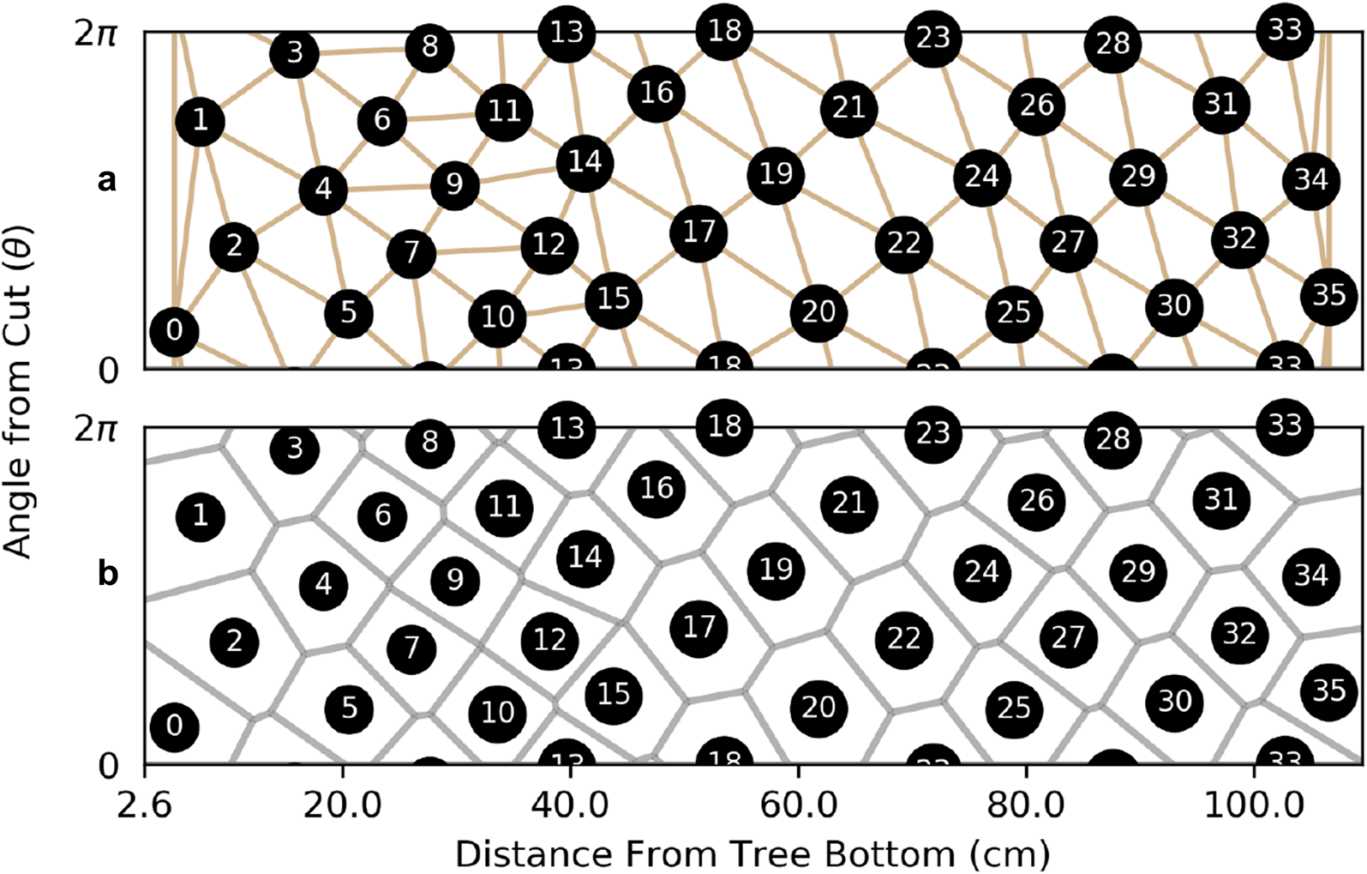
Voronoi analysis of parastichies.** a** The Delaunay triangulation of the nodes on the cylinder reveals contact parastichies, primarily 1-, 2- and 3-parastichies.** b** The Voronoi diagram generates polygons that define primordia

Given a set of points *S* in some space *X*, the *Voronoi diagram* partitions *X* into convex polygons. Each polygon cell and in the diagram contains exactly one point *s* in *S* and all of the points in the cell are closer to *s* than any other point in *S* [23]. The Voronoi diagram can be used to identify contact parastichies by using the Voronoi cells as a proxy for the “shape” of the primordia. Let *S* be the locations of primordia and computed over the surface of a cylinder (a periodic 2D strip). Then the edges in *Delaunay triangulation* (the mathematical dual of the Voronoi diagram) represent contact parastichies based on the relative locations of the primordia. Figure 9 shows the Delaunay triangulation and the Voronoi diagram for the set of nodes in out sample. The most prominent lines are the 1-, 2- and 3-parastichies.

### Insertion order permutations

Mutant plants often exhibit sequences of non-canonical divergence angles due to permutations in the order that primordia form at the shoot apical meristem. These permutations have a genetic basis in* Arabidopsis* by regulating hormone-based inhibitory fields [33, 34]. If noise is limited then insertion order permutations can be detected by patterns (“motifs”) in the sequence of divergence fractions. In particular, if *d* is the divergence fraction of the system, then the motif $[2d, -d, 2d]$ corresponds to a 2-permutation in which 2 adjacent nodes have been swapped. The presence of two 2-permutations adjacent to each other is indicated by the motif $[2d,-d,-3d,2d]$. Higher-order chains of permutations result in other motifs containing multiples of the divergence fraction [33].

**Fig. 10:**
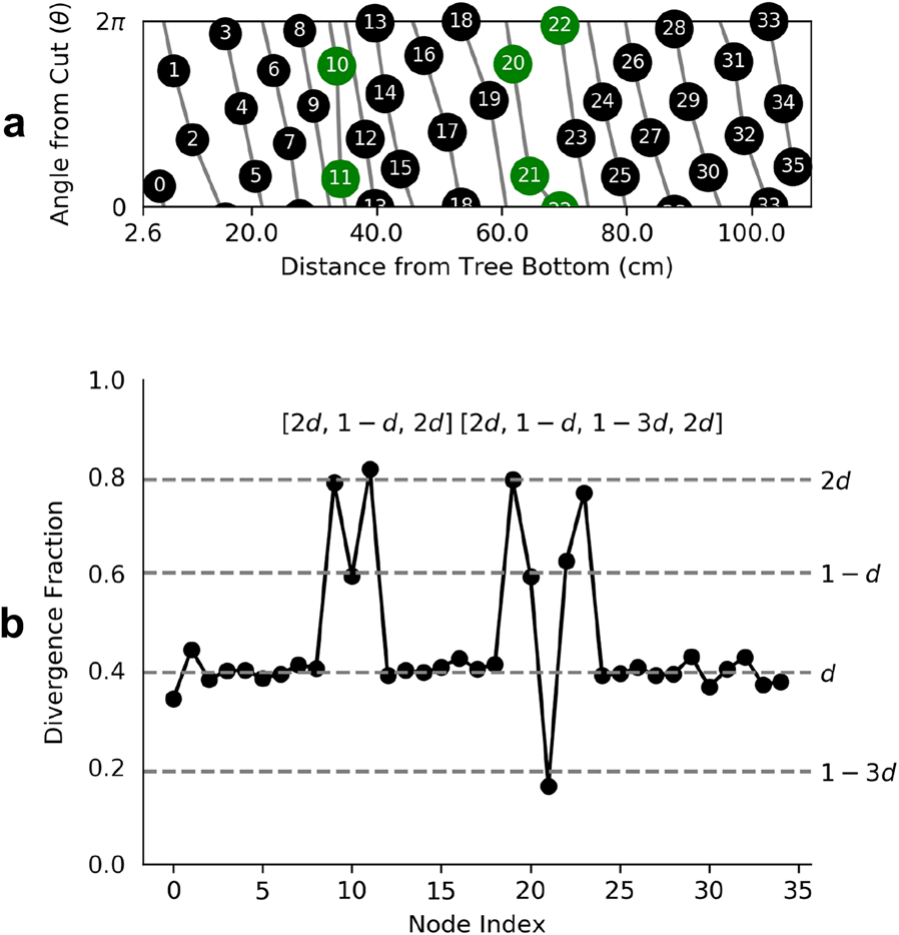
Detecting node insertion permutations.** a** The genetic spiral in the cherry tree nodes with three artificial 2-permutations (shown in green) made by swapping the angle from the cut in pairs of adjacent nodes.** b** Motifs in the succession of divergence angles identify permutations in insertion order. The first motif indicates a 2-permutation and the second motif indicates two 2-permutations that are adjacent to each other

In the case of a normal cherry tree we would not anticipate permutations in the insertion order of organs. However, by artificially swapping the angle measurements between two nodes with adjacent indices we can simulate a 2-permutation. Figure 10 shows three 2-permutations added to our dataset and motifs in the sequence of divergence fractions induced by the insertion order permutations.

## Discussion

**Fig. 11:**
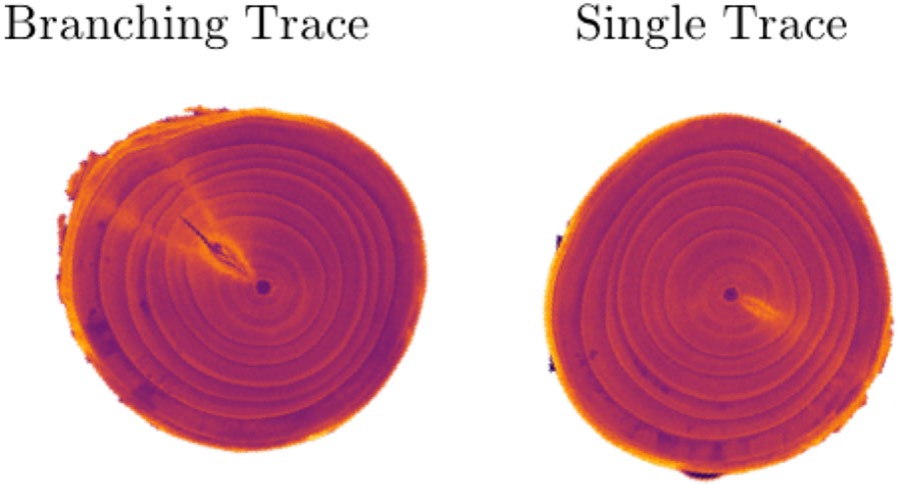
Slices from a X-ray computed tomography (CT) scan of cherry specimen. Like MRI, CT reveals traces as high intensity regions and could be used to study phyllotactic patterns

The 36 nodes detected in our sample were patterned in the tree’s 1st year of growth (2009). The traces connecting to the epicormic buds traverse 7 years of secondary growth. In the orchard, trees are planted as unbranched “whip” of a genetically compound tree, that is, a 1-year-old nursery tree is comprised of the shoot of the scion genotype (fruiting variety) that grew in the nursery the previous year from a bud that was grafted onto a rootstock genotype. A whip nursery tree is typically 1 to 1.5 m tall. In this experiment, the number (8) of annual growth rings at the top of the scanned trunk section matched the number at the bottom; therefore, each node in the section of the scanned trunk originated in the same year, all on the original whip nursery tree. To our knowledge, this is the first accounting of a single growing season’s complement of sweet cherry nodes from origin through 8 years of trunk growth that also identified the persistence of epicormic bud traces. That is, the phyllotactic patterning that occurred during juvenile shoot growth remains in a mature tree, and can be detected using a combination of magnetic resonance imaging and image processing approaches. Figure 11 demonstrates that X-ray computed tomography (CT) also reveals traces as high intensity rays and could be used for a similar analysis.

## Conclusions

Our results have practical implications for orchard rejuvenation and directed approaches to influence tree architecture. As orchards age, yield and fruit quality can begin to decline if fruiting sites become shaded and/or portions of the tree become infected by diseases. Epicormic buds serve as a “bank” for new branches to sprout and rejuvenate orchards. Magnetic resonance imaging provides a clear picture of how full that bank was after 8 years. This could facilitate study of how persistence of epicormic buds may be affected by cultivar, vigor, harsh winters, drought, disease, and spring freezes. Determination of phyllotaxis provides growers with the potential to identify where an epicormic bud is located so that orchard training measures may be attempted to force a branch to sprout at that location. This could help growers fill “holes” in tree canopies to increase fruiting potential and lengthen the life of the orchard. The study of epicormic structures, which are hidden within secondary growth, using tomographic methods also opens the possibility of studying genetic and environmental influences on such structures.

Our results also provide an empirical way to measure phyllotactic parameters, including family of parastichies which capture embedded mathematical information more fully than just the genetic spiral. Much work in phyllotaxy has focused on generative models, but the image processing techniques presented here provide a method to isolate nodes and place them in the context of shoot growth using anatomical features. This is even possible if the features are difficult to discern by eye, or embedded internally within extensive secondary growth. With larger sets of node locations, Fourier methods could further automate the process of finding parastichy numbers [35, 36]. We could further quantify the geometry and topological deviations in a phyllotactic pattern using the “ontogenetic graph”, which is derived from the Voronoi diagram [23, 33]. Isolating features in mature specimens can lead to insights regarding the developmental history of a plant, which is crucial to manipulate plant forms in a directed way and mechanistically understand the origins of morphology. Automated methodology to model imaging features—from MRI or otherwise—into a developmental context is an important first step towards an empirical mathematical framework for measuring plant morphology [33].

## Data Availability

Codes are available on Github (https://github.com/eithun/cherry-phyllotaxy), and raw data are available on the figshare repository (https://doi.org/10.6084/m9.figshare.7409843).

## Abbreviations

MRI: magnetic resonance imaging
CT: computed tomography
